# Comparison of deltoid ligament repair and non-repair in acute ankle fracture: A meta-analysis of comparative studies

**DOI:** 10.1371/journal.pone.0258785

**Published:** 2021-11-12

**Authors:** Wenxuan Guo, Wujie Lin, Wenhuan Chen, Yu Pan, Rujie Zhuang

**Affiliations:** 1 Department of Orthopaedics, The First Affiliated Hospital of Zhejiang Chinese Medical University, Hangzhou, Zhejiang, China; 2 The First Clinical College, Zhejiang Chinese Medical University, Hangzhou, Zhejiang, China; 3 Department of Orthopaedics, Huangyan Hospital of Traditional Chinese Medicine, District Huangyan, Taizhou, Zhejiang, China; 4 Third Clinical Medical School, Guangzhou University of Chinese Medicine, District Baiyun, Guangzhou, Guangdong, China; Assiut University Faculty of Medicine, EGYPT

## Abstract

**Background:**

Rupture of the deltoid ligament (DL) in acute ankle fracture is very common. However, there is still insufficient evidence on whether to repair the DL in acute ankle fracture. Therefore, a systematic review and meta-analysis of comparative studies was performed to report the outcome of DL repair in acute ankle fracture.

**Methods:**

The PubMed, Cochrane Library, EMBASE and Web of Science databases were searched from the inception dates to October 31, 2020, for comparative studies. The methodological quality was evaluated based on the risk-of-bias tool of the Cochrane Collaboration for Randomized Controlled Trials (RCTs) or the Risk-of-Bias Assessment Tool for Non-randomized Studies (RoBANS). The post-operative medial clear space (MCS), final MCS, American Orthopaedic Foot and Ankle Society (AOFAS) ankle-hindfoot score, visual analogue scale (VAS) score and incidence of complications were analysed.

**Results:**

A total of 8 comparative studies involving 388 participants who suffered Weber type B or C ankle fractures were included in this meta-analysis. The results showed that the post-operative MCS, final MCS, AOFAS score and rate of complications were statistically superior in the DL repair group. For the VAS score, there was no significant difference between the DL repair group and the DL non-repair group.

**Conclusions:**

In this meta-analysis of comparative studies, DL repair offered great advantages in terms of the post-operative MCS, final MCS, AOFAS score and rate of complications compared with non-repair. The repair of the DL in patients with acute ankle fractures might be beneficial to ankle joint stability and assist in improving the quality of ankle reduction. More high-quality and prospective studies with long follow-up durations are needed to further demonstrate the superiority of DL repair over non-repair.

## Introduction

Rupture of the deltoid ligament (DL) in acute ankle fracture is very common. The reported incidence of DL tears in patients with ankle fracture is 40% and 58% on arthroscopy and magnetic resonance imaging (MRI), respectively [1, 2]. As a stabilizing component for the medial structure of the ankle joint, the DL consists of superficial and deep layers [3]. The superficial DL resists external rotation of the talus and eversion of the hindfoot. In contrast, the main function of the deep DL is to resist posterior and lateral movement of the talus, as well as prevent valgus angulation [4, 5].

Although the diagnosis of DL rupture is still controversial, most authors agree that a medial clear space (MCS) ≥5 mm on stress radiography is an indication of DL rupture [6, 7]. Some early studies showed no necessity for DL repair because of the lack of a difference in clinical outcomes between non-repair and repair [8, 9]. With further research on the anatomy, physiology and biomechanics of the DL, the contribution of the DL to the medial stability of the ankle joint has received increasing attention [10]. The DL may heal with conservative treatment, but its biomechanical function may not be restored if the ligament is in a prolonged or other abnormal state [11]. Because the deep deltoid ligament is difficult to repair, some authors believe that fixation of the syndesmosis can also stabilize the ankle mortise instead of repair of the DL [10]. Other authors have also used transarticular external fixation rather than DL repair to provide a stable ankle mortise [12]. However, syndesmotic screws and external fixation are not sufficient for direct repair of the ligament. With the widespread use of suture anchors, some studies have reported that this approach is superior and beneficial for repairing the DL [13–15]. There is still insufficient evidence on whether to repair the DL in acute ankle fracture.

Therefore, a systematic review and meta-analysis of comparative studies was performed to report the outcomes of DL repair in acute ankle fracture.

## Methods

This meta-analysis was prepared based on the Preferred Reporting Items for Systematic Reviews and Meta-analyses (PRISMA) guidelines [16]. A PRISMA checklist has been provided in the S1 Checklist.

### Search strategy

We searched the PubMed, Cochrane Library, EMBASE and Web of Science databases from the inception dates to October 31, 2020, using the keywords “ankle”, “malleolus”, “fracture”, “deltoid”, and “medial collateral ligament”. In addition, we screened the reference lists of the included studies for additional relevant studies.

### Selection criteria

Studies were selected based on the following inclusion criteria: (1) a target population of adults over 16 years old with acute ankle fractures; (2) clinical trials comparing surgical repair of the DL versus fixation of the syndesmosis or non-operative treatment; and (3) trials reporting the MCS or the American Orthopaedic Foot and Ankle Society (AOFAS) ankle-hindfoot score as one of the primary outcomes. The exclusion criteria were as follows: (1) conference abstracts; (2) trials without available data; and (3) studies not written in English.

### Study selection and data extraction

Two independent researchers (W.-X.G., W.-J.L.) screened the study titles and abstracts according to the inclusion criteria. The full text of the studies potentially meeting the eligibility criteria were retrieved for a more detailed read to make a final decision regarding inclusion. The following data were extracted: lead author; publication year; country of origin; study design; sample size; age; fracture type; repair technique; outcome measures; and follow-up duration.

### Quality assessment

Two independent investigators (W.-X.G., W.-J.L.) evaluated the quality of the included studies. The risk-of-bias tool of the Cochrane Collaboration for Randomized Controlled Trials (RCTs) was used by two independent reviewers to assess the methodological quality [17]. The 7 items used to evaluate bias in each trial included the randomization sequence generation, allocation concealment, blinding of participants and personnel, blinding of outcome assessments, incomplete outcome data, selective reporting, and other biases, such as the baseline characteristics between different groups. The methodological quality of the non-randomized studies was assessed using the Risk-of-Bias Assessment Tool for Non-randomized Studies (RoBANS) [18]. The 6 items used to evaluate bias for non-randomized studies included the selection of participants, confounding variables, intervention measurements, blinding of outcome assessments, incomplete outcome data, and selective outcome reporting. The level of evidence was assessed according to the Oxford Centre for Evidence-based Medicine Levels of Evidence.

### Data analysis

All meta-analyses were conducted via Review Manager software (RevMan version 5.4, Cochrane Collaboration). The mean difference (MD) was used as the effect analysis statistic for continuous variables; the risk ratio (RR) was used as the effect analysis statistic for categorical variables. The 95% confidence interval (CI) was calculated for each statistic. Statistical heterogeneity among summary data was evaluated using the I^2^ statistic. If I^2^≤50%, the heterogeneity was not significantly different, and a fixed-effects model was used for the meta-analysis. If there was statistical heterogeneity among studies, the source of heterogeneity was further analysed. After excluding the obvious source of clinical heterogeneity, a random-effects model was used to pool the data. When obvious clinical heterogeneity existed, the researchers performed subgroup or sensitivity analyses or only descriptive analyses. Study-specific and pooled estimates are graphically depicted by forest plots. P <0.05 was considered statistically significant.

## Results

**Fig 1:**
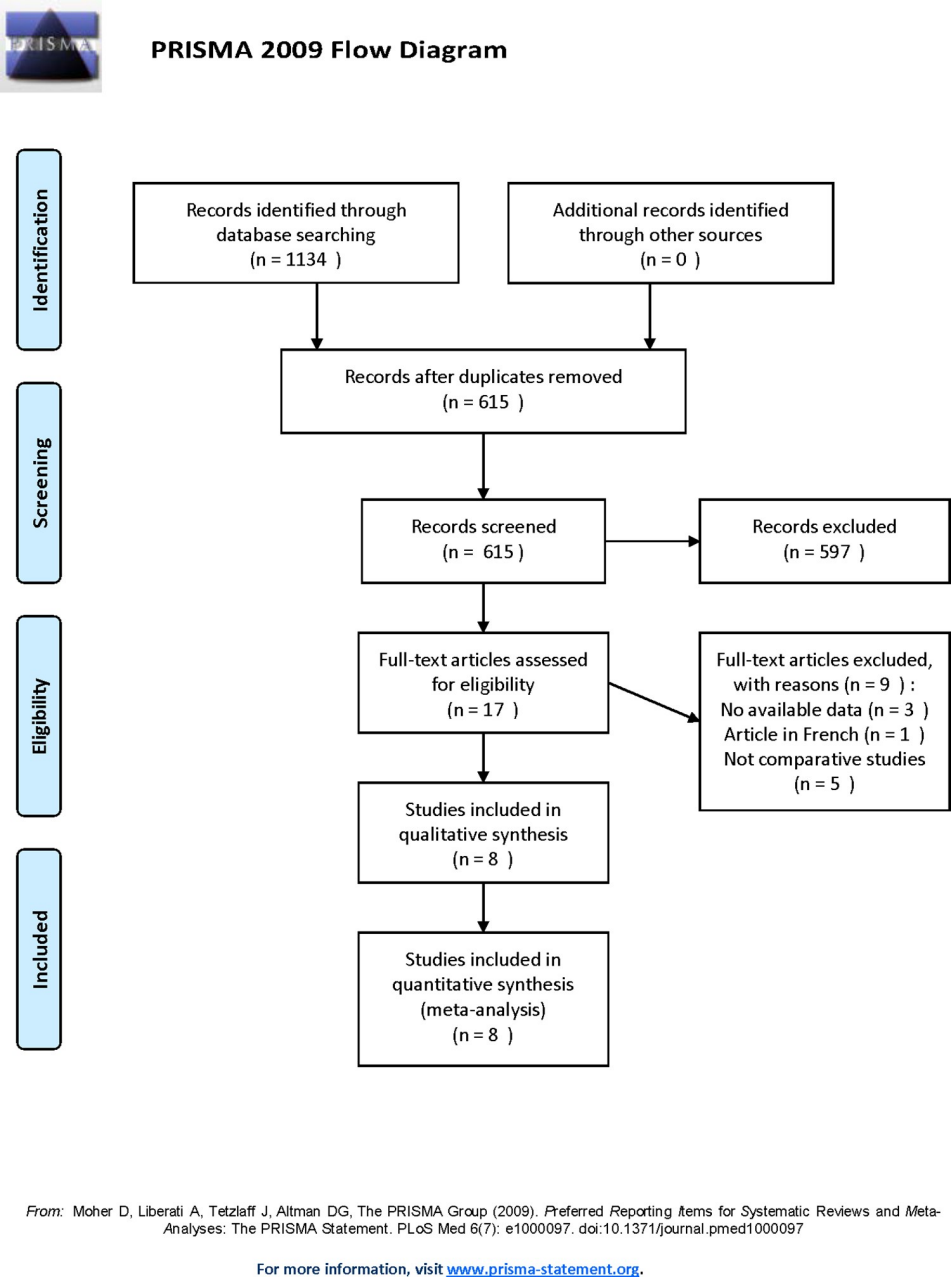
Flow diagram of study searching and selection process.

From the searches for published comparative studies, 1134 potentially eligible records were identified, and 17 studies were reviewed in full text. Of these studies, 8 trials [10, 12, 15, 19–23] met the inclusion criteria and were retained, while the others were excluded for various reasons (Fig 1).

### Study characteristics

The characteristics of the studies analysed are presented in Table 1. Among the included studies, 2 trials were randomized controlled trials [10, 20], 1 study was a prospective cohort study [22], and 5 studies were non-randomized comparative studies [12, 15, 19, 21, 23]. Five studies [15, 19, 20, 22, 23] compared acute ankle fractures with or without DL repair, 2 trials [10, 21] compared DL repair and syndesmotic fixation in bimalleolar equivalent ankle fractures, and 1 trial [12] compared transarticular external fixation and DL repair. A total of 388 participants who suffered Weber type B or C ankle fractures were included in this meta-analysis; among them, 191 ankles received operative treatment for the DL, 135 ankles received conservative treatment for the DL, 42 ankles underwent syndesmotic fixation with screws, and 20 ankles underwent transarticular external fixation. The mean age of the patients ranged from 35.2 to 41.6 years. The mean follow-up period ranged from 13.1 to 53.7 months. The post-operative MCS was reported in 4 trials [15, 19, 22, 23], and the final MCS was reported in 5 studies [15, 19, 20, 22, 23]. Six studies [10, 12, 15, 21–23] evaluated the AOFAS ankle-hindfoot score, and 5 studies [10, 12, 15, 21, 23] evaluated the visual analogue scale (VAS) score. Complications, including malreduction and medial side pain, were reported in all included trials.

**Table 1:** Baseline characteristics of the included studies.

Included Studies	Country	Study design	Sample size	Mean age (years)	Fracture type	Outcomes	Mean follow-up (months)
Choi 2020	Korea	Retrospective	Repair: 19	38.4	Weber type B	①②⑤	13.6
			Non-repair: 15				
Gu 2017	China	Prospective	Repair: 20	39.1	Not Reported	②⑤	13.1
			Non-repair: 20				
Jones 2015	USA	Retrospective	Repair: 12	39.0	Weber type B	③④⑤	50.3
			Syndesmotic fixation: 15				
Li 2019	China	Retrospective	Repair: 23	39.4	Weber type B	③④⑤	27.2
			Transarticular external fixation: 20				
Sun 2018	China	Prospective cohort study	Repair: 28	35.2	Weber type B	①②③⑤	41.7
			Non-repair: 13				
Woo 2017	Korea	Retrospective	Repair: 41	40.6	Weber type B and C	①②③④⑤	17
			Non-repair: 37				
Wu 2018	China	Randomized controlled trial	Repair: 24	39.6	Weber type B and C	③④⑤	23.1
			Syndesmotic fixation: 27				
Zhao 2017	China	Retrospective	Repair: 20	39.5	Weber type B and C	①②③④⑤	53.7
			Non-repair: 54				

①Post-operative MCS; ②Final MCS; ③AOFAS: American Orthopaedic Foot and Ankle Society (AOFAS) ankle-hindfoot score; ④VAS: The visual analogue scale; ⑤Complication.

### Risk-of-bias assessments

#### Randomized controlled trials

**Fig 2:**
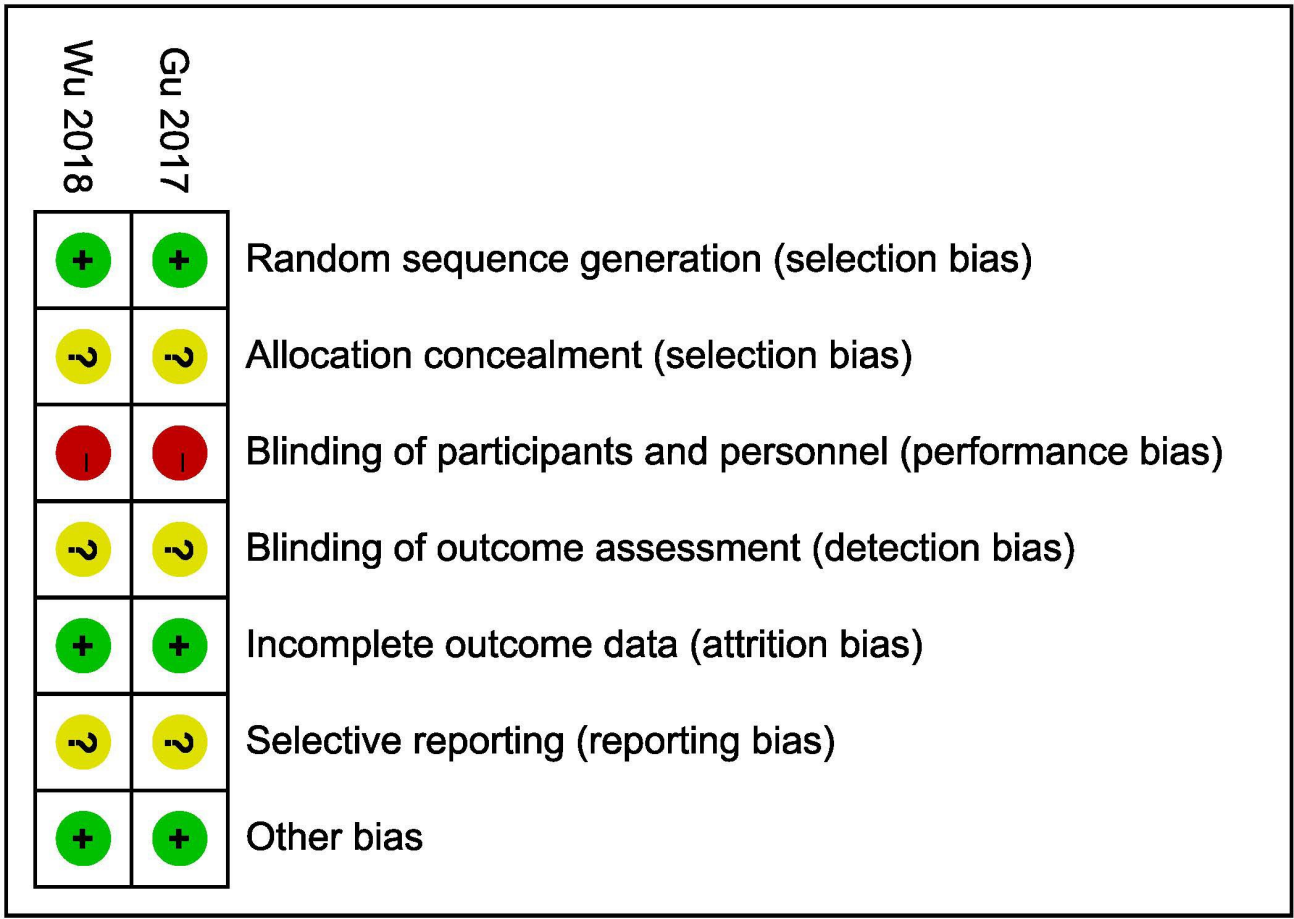
The methodological quality for RCTs.

Only 2 studies [10, 20] were described as RCTs. The methodological quality of the RCTs according to the Cochrane Collaboration risk-of-bias criteria is shown in Fig 2. No trials reported the methods for allocation concealment. Blindness was difficult to achieve for the participants and personnel because of the nature of the operative interventions. Blindness for outcome assessments was not reported in either trial. The study protocols were not found, so it was difficult to assess the reporting bias. Level 1b evidence was observed for the included RCTs based on the Oxford Centre for Evidence-based Medicine Levels of Evidence.

#### Non-randomized comparative studies

**Fig 3:**
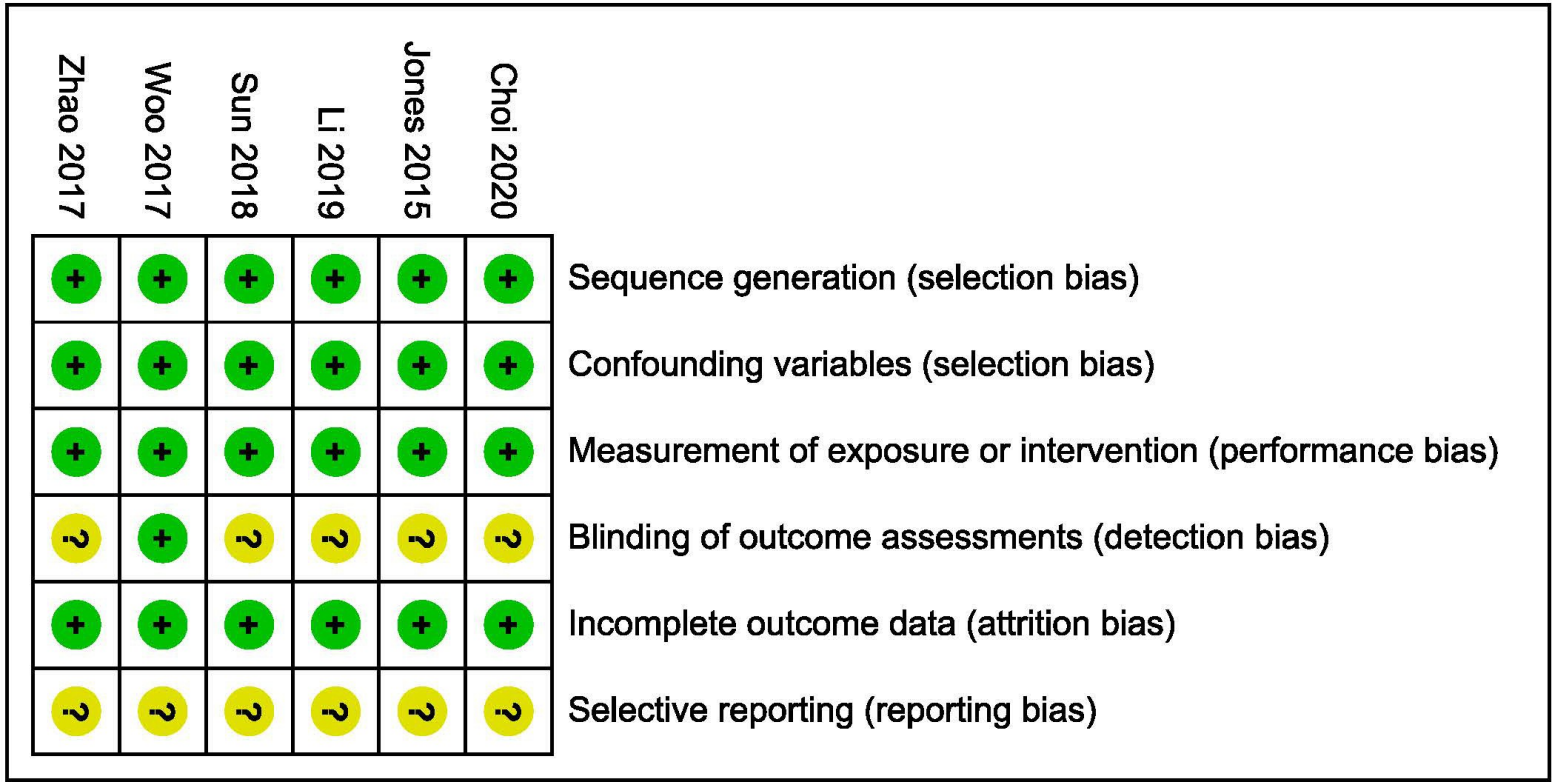
The methodological quality for non-randomized comparative studies.

The bias of the prospective cohort study and non-randomized comparative study was assessed by the RoBANS (Fig 3). One study [23] reported blinding of the observers to the clinical information of the patients. The protocols were not found in all studies, so it was unclear whether the published report included all expected outcomes. Level 2b evidence was observed for all included studies based on the Oxford Centre for Evidence-based Medicine Levels of Evidence.

### Meta-analysis results

#### Post-operative MCS

**Fig 4:**
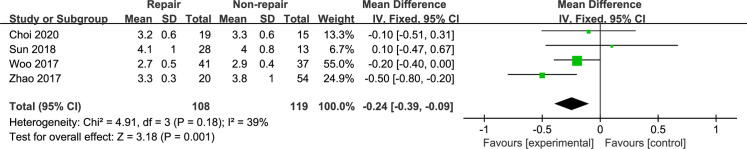
The forest plot of post-operative MCS.

The post-operative MCS was reported in 4 included studies [15, 19, 22, 23]. Meta-analysis with the fixed-effects model (Fig 4) showed that the post-operative MCS was statistically superior in the DL repair group (MD, −0.24 [95% CI, −0.39, −0.09]), with moderate heterogeneity (I^2^: 39%).

#### Final MCS

**Fig 5:**
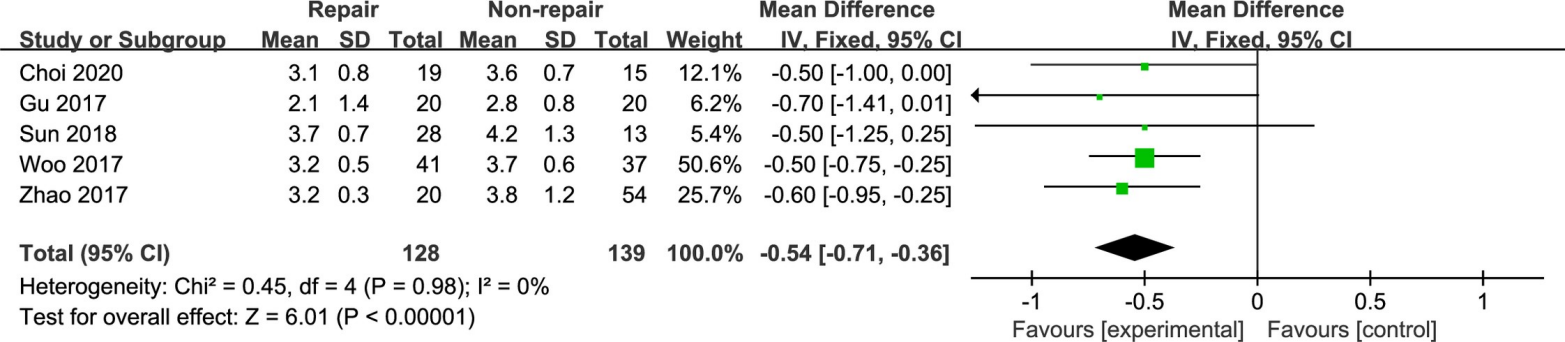
The forest plot of final MCS.

The final MCS was reported in 5 included studies [15, 19, 20, 22, 23]. Meta-analysis with the fixed-effects model (Fig 5) showed that the final MCS was statistically superior in the DL repair group (MD, −0.54 [95% CI, −0.71, −0.36]), with no heterogeneity (I^2^: 0%).

#### AOFAS ankle-hindfoot score

**Fig 6:**
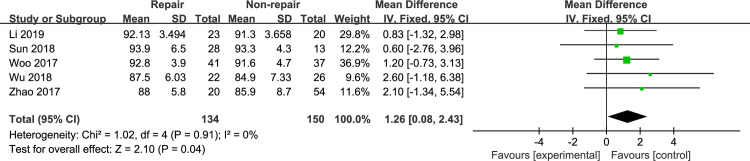
The forest plot of AOFAS ankle-hindfoot score.

**Fig 7:**
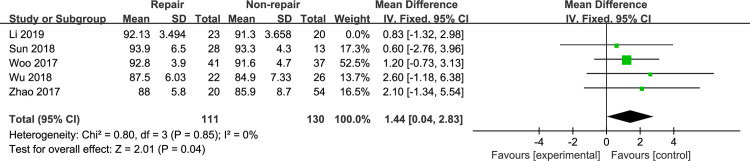
The forest plot of AOFAS ankle-hindfoot score after sensitivity analysis.

The AOFAS ankle-hindfoot score was reported in 6 included studies [10, 12, 15, 21–23]. Jones et al [21] reported that the mean AOFAS score was 12.7 and 14 in the DL repair group and the syndesmotic fixation group, respectively. According to the other clinical outcomes reported in this article, we considered this score to be unreasonable, so we excluded this article from our analysis. Meta-analysis with the fixed-effects model (Fig 6) showed that the AOFAS ankle-hindfoot score was significantly superior in the DL repair group (MD, 1.26 [95% CI, 0.08, 2.43]), with no heterogeneity (I^2^: 0%). Li et al [12] reported the outcomes of patients treated with external fixation. A sensitivity analysis was performed that excluded the studies by Li et al [12], and the results remained unchanged (Fig 7).

#### Pain score

**Fig 8:**
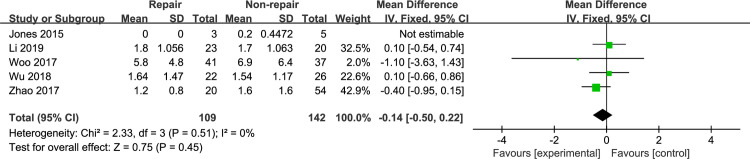
The forest plot of pain score.

The VAS score was reported in 5 studies [10, 12, 15, 21, 23]. Meta-analysis with the fixed-effects model (Fig 8) showed no significant difference between the DL repair group and the DL non-repair group (MD, −0.14 [95% CI, −0.50, 0.22]), with no heterogeneity (I^2^: 0%).

#### Complications

**Fig 9:**
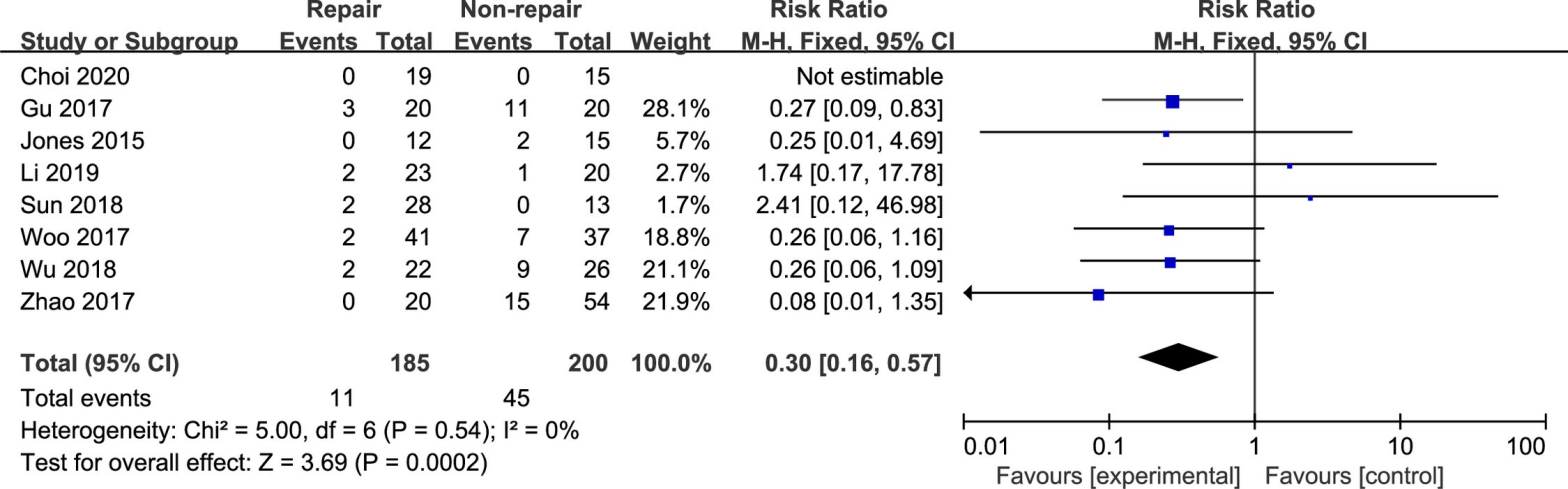
The forest plot of complications.

The total complications were reported in all included studies [10, 12, 15, 19–23]. Meta-analysis with the fixed-effects model (Fig 9) showed that the rate of complications was significantly higher in the DL non-repair group than in the DL repair group (RR, 0.30 [95% CI, 0.16, 0.57]), with no heterogeneity (I^2^: 0%).

## Discussion

There is no consensus regarding the optimal treatment of DL rupture, but a biomechanical study confirmed that DL repair enhances ankle stability in ankle fractures with both syndesmotic and deltoid disruption [24]. A lack of meta-analyses and high-quality studies assessing the reduction of ankle fractures and outcomes of DL repair make it difficult to determine whether the necessity for and efficacy of DL repair surpass those of conservative treatment. In this meta-analysis, we found that DL repair had significantly superior outcomes in terms of the post-operative MCS, final MCS, AOFAS ankle-hindfoot score and rate of complications compared to conservative interventions. Sensitivity analyses that excluded trials that enrolled patients to compare DL repair with syndesmotic fixation or transarticular external fixation did not alter these results.

An obvious increase in the MCS on positive stress examination is frequently an indication of DL rupture [25]. Meanwhile, the MCS is associated with deltoid dysfunction, ankle instability, and clinical outcomes after ankle fracture [26]. Zhao et al [15] compared the outcomes of DL repair and non-repair in 74 patients over a mean follow-up of 53.7. The author reported that DL repair significantly decreases the post-operative MCS. Gu et al [20] reported that DL reconstruction played a positive role in restoring the MCS. In our meta-analysis, we found significantly superior results in terms of both the post-operative MCS and the final MCS in the DL repair group. Some authors consider that even small deviations from anatomical alignment can result in a significant reduction in the joint contact area [23]. Therefore, restoration of the MCS will allow reconstruction of the ankle mortise, stabilize the ankle joint and delay the onset of arthritis.

The AOFAS ankle-hindfoot score is widely used to assess clinical outcomes in patients with ankle fractures [27–29]. Woo et al [23] performed a retrospective comparative study to assess the clinical outcomes and radiologic findings in 78 patients over a mean follow-up of 17 months. There was no significant difference in the AOFAS score between the DL repair group and the non-repair group. Wu et al [10] performed an RCT to evaluate clinical outcomes in the syndesmotic fixation group and the DL repair group. The AOFAS score showed no significant difference between the groups, which is consistent with Woo’s results. However, some recent case series have suggested better outcomes after DL repair [13, 14]. Our meta-analysis showed a significant difference in the AOFAS score between the DL repair group and the non-repair group, and sensitivity analyses did not change these results. Although there was no significant difference in the previously included comparative studies, slightly better results could be seen in the DL repair group than in the non-repair group. In addition, the sample size of each study was small, which could have prevented the detection of a significant difference. When the data were combined for meta-analysis, with the increase in sample size, the results showed significant differences. The pain score showed no significant difference in our meta-analysis, but the AOFAS ankle-hindfoot score was also use to assess clinical outcomes, such as joint function and range of motion. Hsu et al [13] reported the outcomes of DL repair in 14 National Football League (NFL) players. These players returned to the game for an average of 1.6 seasons without complaining of complications. Similarly, patients treated with DL repair may have better functional outcomes in our study, as the AOFAS score was statistically superior in the DL repair group even though there was no significant difference in the VAS score between the two groups.

Zhao et al [15] reported a high malreduction rate in the DL non-repair group, especially in cases of Weber type C fracture. The total complication rate was significantly higher in the DL non-repair group in our meta-analysis. The malreduction rate accounts for a large proportion of complications. Hintermann et al [30] believed that if Weber type C ankle fractures cannot be completely stabilized after internal fixation, the medial ankle ligament should be reconstructed carefully. Zhao et al [15] found DL repair could be performed in patients without malreduction, even in patients with Weber type C fractures. Similarly, Mococain et al [24] reported that deltoid ligament repair can enhance the stability of ankle fractures with both syndesmotic and deltoid ruptures in a biomechanical study. Theoretically, non-anatomic deltoid ligament healing may result in instability, persistent medial gutter pain and loss of function, with a risk of early osteoarthritis [31]. Woo et al [23] noted that without DL repair, patients occasionally suffered from persistent medial pain around the DL even after anatomical healing. Some authors believe DL repair will prolong the operation and increase the incidence of wound-related complications [32], and our data analysis shows the same results.

A systematic review and analysis by Dabash et al [33] reported that there may be some benefits to performing DL repair in patients with high fibular fractures, perhaps in combination with syndesmotic fixation. However, the report by Dabash et al [33] included 5 studies and a descriptive analysis. A meta-analysis by Salameh et al [32] reported improvements in the early and late MCS and pain scores. This meta-analysis only included 3 studies and 192 patients, the small sample size and heterogeneity of included studies might not be able to support their conclusion. In addition, the functional outcome of AOFAS did not show any difference between the two groups in this meta-analysis. In our study, DL repair group showed great advantages in terms of the post-operative MCS, final MCS, AOFAS score and rate of complications. To our knowledge, our report is the first meta-analysis to show significant differences in clinical outcomes.

This study still has several limitations. First, due to the exclusion of articles not written in English, some articles may have been omitted. Second, we did not assess the influence of interventions with and without DL repair based on the fracture classification. Third, the mean duration of follow-up varied from 13.1 to 53.7 months. The relatively short duration of follow-up limited the current study because it is known that long-term follow-up is necessary to determine the longevity of the repair technique and the complication rate of ankle osteoarthritis. Fourth, the moderate heterogeneity was observed in post-operative MCS(I^2^:39%). The source of heterogeneity might be from follow-up periods, complications criteria, classification of fracture and operation technique. Therefore, the meta-analysis of post-operative MCS with the random-effects model was performed. We found the results did not change. Last, of the 8 studies included, only 2 were RCTs, which were of poor quality; for example, unclear allocation concealment was used. Therefore, RCTs or prospective studies are needed to reinforce the evidence on the best treatment recommendations for patients with acute ankle fractures and DL rupture.

## Conclusions

In this meta-analysis of comparative studies, DL repair offered great advantages in terms of the post-operative MCS, final MCS, AOFAS score and rate of complications compared with no DL repair. The repair of the DL in patients with acute ankle fractures might be beneficial to ankle joint stability and assist in improving the quality of ankle reduction. More high-quality and prospective studies with long follow-up durations are needed to further demonstrate the superiority of DL repair over non-repair.

## Supporting information

S1 Checklist PRISMA checklist. (DOCX)

S1 File The baseline characteristics of the included studies. (DOCX)

S2 File Flow diagram of the searching processes. (DOC)

S3 File Search strategies in PubMed. (DOCX)

S4 File The methodological quality for included studies. (DOCX)
