# Supplementary material for: Comparison of deltoid ligament repair and non-repair in acute ankle fracture: A meta-analysis of comparative studies
Source: PLoS One. 2021 Nov 12;16(11):e0258785. doi: 10.1371/journal.pone.0258785 (PMC8589189; doi:10.1371/journal.pone.0258785)
Supplement: S1 File — (DOCX) [file pone.0258785.s002.docx]

The baseline characteristics of the included studies

The baseline characteristics of the included studies

| Included Studies | Country | Study design | Sample size | Mean age (years) | Fracture types | Outcomes | Mean Follow-up (months) |
| --- | --- | --- | --- | --- | --- | --- | --- |
| Choi 2020 | Korea | Retrospective | Repair: 19  Non-repair: 15 | 38.4 | Weber type B | ①②⑤ | 13.6 |
| Gu 2017 | China | Prospective | Repair: 20  Non-repair: 20 | 39.1 | Not Reported | ②⑤ | 13.1 |
| Jones 2015 | USA | Retrospective | Repair: 12  Syndesmosis Fixation: 15 | 39.0 | Weber type B | ③④⑤ | 50.3 |
| Li 2019 | China | Retrospective | Repair: 23  Transarticular external fixation: 20 | 39.4 | Weber type B | ③④⑤ | 27.2 |
| Sun 2018 | China | Prospective cohort study | Repair: 28  Non-repair: 13 | 35.2 | Weber type B | ①②③⑤ | 41.7 |
| Woo 2017 | Korea | Retrospective | Repair: 41  Non-repair: 37 | 40.6 | Weber type B and C | ①②③④⑤ | 17 |
| Wu 2018 | China | Randomized controlled trial | Repair: 24  Syndesmosis fixation: 27 | 39.6 | Weber type B and C | ③④⑤ | 23.1 |
| Zhao 2017 | China | Retrospective | Repair: 20  Non-repair: 54 | 39.5 | Weber type B and C | ①②③④⑤ | 53.7 |

①Post-operative MCS; ②Final MCS; ③AOFAS: the American Orthopaedic Foot and Ankle Society (AOFAS) ankle-hindfoot score; ④VAS: the visual analogue scale; ⑤Complication.
