# Supplementary material for: Comparison of deltoid ligament repair and non-repair in acute ankle fracture: A meta-analysis of comparative studies
Source: PLoS One. 2021 Nov 12;16(11):e0258785. doi: 10.1371/journal.pone.0258785 (PMC8589189; doi:10.1371/journal.pone.0258785)
Supplement: S2 File — (DOC) [file pone.0258785.s003.doc]

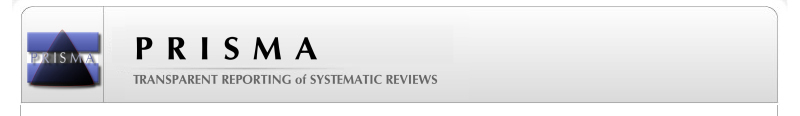
**PRISMA 2009 Flow Diagram**

**Screening**

**Included**

**Eligibility**

**Identification**

Records identified through database searching
(n = 1134 )

Additional records identified through other sources
(n = 0 )

Records after duplicates removed
(n = 615 )

Records screened
(n = 615 )

Records excluded
(n = 597 )

Full-text articles assessed for eligibility
(n = 17 )

Full-text articles excluded, with reasons (n = 9 ) :

No available data (n = 3 )

Article in French (n = 1 )

Not comparative studies (n = 5 )

Studies included in qualitative synthesis
(n = 8 )

Studies included in quantitative synthesis (meta-analysis)
(n = 8 )
