# Supplementary material for: Comparison of deltoid ligament repair and non-repair in acute ankle fracture: A meta-analysis of comparative studies
Source: PLoS One. 2021 Nov 12;16(11):e0258785. doi: 10.1371/journal.pone.0258785 (PMC8589189; doi:10.1371/journal.pone.0258785)
Supplement: S3 File — (DOCX) [file pone.0258785.s004.docx]

Search strategies in PubMed

| Search number | Query | Search Details | Results |
| --- | --- | --- | --- |
| #1 | ankle | "ankle"[MeSH Terms] OR "ankle"[All Fields] OR "ankle joint"[MeSH Terms] OR ("ankle"[All Fields] AND "joint"[All Fields]) OR "ankle joint"[All Fields] OR "ankles"[All Fields] OR "ankle s"[All Fields] | 77,604 |
| #2 | malleolus | "malleolus"[All Fields] | 2,577 |
| #3 | fracture | "fractur"[All Fields] OR "fractural"[All Fields] OR "fracture s"[All Fields] OR "fractures, bone"[MeSH Terms] OR ("fractures"[All Fields] AND "bone"[All Fields]) OR "bone fractures"[All Fields] OR "fracture"[All Fields] OR "fractured"[All Fields] OR "fractures"[All Fields] OR "fracturing"[All Fields] | 317,622 |
| #4 | deltoid | "deltoid muscle"[MeSH Terms] OR ("deltoid"[All Fields] AND "muscle"[All Fields]) OR "deltoid muscle"[All Fields] OR "deltoid"[All Fields] OR "deltoids"[All Fields] | 5,203 |
| #5 | medial collateral ligament | ("medial"[All Fields] OR "mediale"[All Fields] OR "mediales"[All Fields] OR "medialisation"[All Fields] OR "medialise"[All Fields] OR "medialised"[All Fields] OR "medialization"[All Fields] OR "medializations"[All Fields] OR "medialize"[All Fields] OR "medialized"[All Fields] OR "medializes"[All Fields] OR "medializing"[All Fields] OR "medially"[All Fields] OR "medials"[All Fields]) AND ("collateral ligaments"[MeSH Terms] OR ("collateral"[All Fields] AND "ligaments"[All Fields]) OR "collateral ligaments"[All Fields] OR ("collateral"[All Fields] AND "ligament"[All Fields]) OR "collateral ligament"[All Fields]) | 3,544 |
| #6 | #1 OR #2 | "ankle"[MeSH Terms] OR "ankle"[All Fields] OR "ankle joint"[MeSH Terms] OR ("ankle"[All Fields] AND "joint"[All Fields]) OR "ankle joint"[All Fields] OR "ankles"[All Fields] OR "ankle s"[All Fields] OR "malleolus"[All Fields] | 78,460 |
| #7 | #3 AND #6 | ("fractur"[All Fields] OR "fractural"[All Fields] OR "fracture s"[All Fields] OR "fractures, bone"[MeSH Terms] OR ("fractures"[All Fields] AND "bone"[All Fields]) OR "bone fractures"[All Fields] OR "fracture"[All Fields] OR "fractured"[All Fields] OR "fractures"[All Fields] OR "fracturing"[All Fields]) AND ("ankle"[MeSH Terms] OR "ankle"[All Fields] OR "ankle joint"[MeSH Terms] OR ("ankle"[All Fields] AND "joint"[All Fields]) OR "ankle joint"[All Fields] OR "ankles"[All Fields] OR "ankle s"[All Fields] OR "malleolus"[All Fields]) | 12,510 |
| #8 | #4 OR #5 | "deltoid muscle"[MeSH Terms] OR ("deltoid"[All Fields] AND "muscle"[All Fields]) OR "deltoid muscle"[All Fields] OR "deltoid"[All Fields] OR "deltoids"[All Fields] OR (("medial"[All Fields] OR "mediale"[All Fields] OR "mediales"[All Fields] OR "medialisation"[All Fields] OR "medialised"[All Fields] OR "medialization"[All Fields] OR "medializations"[All Fields] OR "medialize"[All Fields] OR "medialized"[All Fields] OR "medializes"[All Fields] OR "medializing"[All Fields] OR "medially"[All Fields] OR "medials"[All Fields]) AND ("collateral ligaments"[MeSH Terms] OR ("collateral"[All Fields] AND "ligaments"[All Fields]) OR "collateral ligaments"[All Fields] OR ("collateral"[All Fields] AND "ligament"[All Fields]) OR "collateral ligament"[All Fields])) | 8,688 |
| #9 | #7 AND #8 | ("fractur"[All Fields] OR "fractural"[All Fields] OR "fracture s"[All Fields] OR "fractures, bone"[MeSH Terms] OR ("fractures"[All Fields] AND "bone"[All Fields]) OR "bone fractures"[All Fields] OR "fracture"[All Fields] OR "fractured"[All Fields] OR "fractures"[All Fields] OR "fracturing"[All Fields]) AND ("ankle"[MeSH Terms] OR "ankle"[All Fields] OR "ankle joint"[MeSH Terms] OR ("ankle"[All Fields] AND "joint"[All Fields]) OR "ankle joint"[All Fields] OR "ankles"[All Fields] OR "ankle s"[All Fields] OR "malleolus"[All Fields]) AND ("deltoid muscle"[MeSH Terms] OR ("deltoid"[All Fields] AND "muscle"[All Fields]) OR "deltoid muscle"[All Fields] OR "deltoid"[All Fields] OR "deltoids"[All Fields] OR (("medial"[All Fields] OR "mediale"[All Fields] OR "mediales"[All Fields] OR "medialisation"[All Fields] OR "medialise"[All Fields] OR "medialised"[All Fields] OR "medialization"[All Fields] OR "medializations"[All Fields] OR "medialize"[All Fields] OR "medialized"[All Fields] OR "medializes"[All Fields] OR "medializing"[All Fields] OR "medially"[All Fields] OR "medials"[All Fields]) AND ("collateral ligaments"[MeSH Terms] OR ("collateral"[All Fields] AND "ligaments"[All Fields]) OR "collateral ligaments"[All Fields] OR ("collateral"[All Fields] AND "ligament"[All Fields]) OR "collateral ligament"[All Fields]))) | 344 |
