# Supplementary material for: Comparison of deltoid ligament repair and non-repair in acute ankle fracture: A meta-analysis of comparative studies
Source: PLoS One. 2021 Nov 12;16(11):e0258785. doi: 10.1371/journal.pone.0258785 (PMC8589189; doi:10.1371/journal.pone.0258785)
Supplement: S4 File — (DOCX) [file pone.0258785.s005.docx]

The methodological quality for RCTs.

| Study | Random sequence generation | Allocation concealment | Blinding of participants and personnel | Blinding of outcome assessment | Incomplete outcome data | Selective reporting | Other bias | | Level of  evidence | |
| --- | --- | --- | --- | --- | --- | --- | --- | --- | --- | --- |
| Gu 2017 | Low risk | Unclear risk | High risk | Unclear risk | Low risk | Unclear risk | | Low risk | | 1b |
| Wu2018 | Low risk | Unclear risk | High risk | Unclear risk | Low risk | Unclear risk | | Low risk | | 1b |

The methodological quality for non-randomized comparative studies.

| Study | Sequence generation | Confounding variables | Measurement of exposure or intervention | Blinding of outcome assessments | Incomplete outcome data | Selective reporting | Level of  evidence |
| --- | --- | --- | --- | --- | --- | --- | --- |
| Choi 2020 | Low risk | Low risk | Low risk | Unclear risk | Low risk | Unclear risk | 2b |
| Jones 2015 | Low risk | Low risk | Low risk | Unclear risk | Low risk | Unclear risk | 2b |
| Li 2019 | Low risk | Low risk | Low risk | Unclear risk | Low risk | Unclear risk | 2b |
| Sun 2018 | Low risk | Low risk | Low risk | Unclear risk | Low risk | Unclear risk | 2b |
| Woo 2017 | Low risk | Low risk | Low risk | Low risk | Low risk | Unclear risk | 2b |
| Zhao 2017 | Low risk | Low risk | Low risk | Unclear risk | Low risk | Unclear risk | 2b |
